# Cardiac Epithelioid PEComa: Report of Two Cases and Review of the Literature

**DOI:** 10.1155/2012/521678

**Published:** 2012-09-03

**Authors:** Huilin Niu, F. W. Wang, Paul J. Zhang, Zhanyong Bing

**Affiliations:** ^1^Department of Pathology, Guangzhou Children's Hospital, Guangzhou 510120, China; ^2^Department of Pathology and Laboratory Medicine, Hospital of the University of Pennsylvania, 3400 Spruce Street, Philadelphia, PA 19104, USA

## Abstract

Cardiac PEComa is very rare. We reported two cases of epithelioid PEComas, one in an adult and one in a 2-year-old child. Both tumors were composed of sheets of epithelioid cells with coagulation necrosis. In addition, the adult case showed marked nuclear atypia and high mitotic activity with atypical mitosis and the pediatric case showed unusual clear cell features. Immunohistochemically, both tumors were positive for HMB-45 and SMA and negative for S100 and cytokeratin. Electron microscopy was performed in the pediatric case and showed premelanosomes. The adult patient developed extensive metastasis indicating malignant behavior. Prior to the two cases, only 5 other cases of cardiac PEComa were reported and the literatures are reviewed.

## 1. Introduction

PEComa was first proposed by Bonetti et al. [1] and is a mesenchymal tumor composed of perivascular epithelioid cell (PEC) and includes lymphangiomyomatosis, clear cell “sugar” tumor of the lung, and angiomyolipoma of the kidney and morphologically and immunohistochemically related tumors at other sites [2–12]. Epithelioid PEComa is a rare variant, and majority of the cases arise from kidney (also called epithelioid angiomyolipoma, EAML). Radiologically, pure EAML presents as a mass devoid of fat density and is larger than the classical AML. Histologically, pure form of EAML is composed of polygonal cells of perivascular/myoid type without prominent dysmorphic blood vessels and adipose tissue. EAML appears to have more aggressive behavior. These tumor cells have clear to eosinophilic cytoplasm and round-to-oval nuclei with variable nuclear atypia. In addition, other worrisome histological features including coagulative necrosis, mitosis including rare atypical mitosis, may be present [13]. The epithelioid morphology in combination of cytological atypia causes diagnostic difficulty. Only 5 cases of primary PEComas arising from heart have been reported in literatures, 4 in young adults, and one in a 10-year old girl. In this study, we reported two cases of cardiac epithelioid PEComas, one in a 33-year old female and one in a 2-year-old girl.

## 2. Material and Method

The specimen was fixed in a 10% neutral-buffered formalin solution and processed as a routine surgical pathology specimen. Representative sections were embedded in paraffin, processed in the usual manner, and stained with hematoxylin-eosin.

### 2.1. Immunohistochemistry

Immunohistochemical stains were performed on formalin-fixed, paraffin-embedded 4 um tissue sections with the avidin-biotin immunoperoxidase complex method (LSAB2 system, Dako Corporation, Carpinteria, CA, USA) with diaminobenzidine as the chromogen and hematoxylin as the nuclear counterstain. Information about the antibodies used was summarized in Table 1. Antigens were retrieved from the tissue by incubating the tissue sections in a Black and Decker Vegetable Steamer for 20 minutes in Target Retrieval Solution (Dako) preheated to 99°C. The negative control was performed by substituting the primary antibody with nonimmune mouse or rabbit serum. Approximate positive controls were used.

## 3. Clinical History

### 3.1. Case  1

A 2-year-old girl presented with dyspnea and poor sleep for 10 days. Physical examination showed that the patient had cyanosis around her lips, decreased movements, edema of lower extremities, and hypourocrinia. The patient had no fever and her growth and development were normal. Cardiovascular system examination revealed lower heart sounds with irregular rate (heart rate 200 times/minute), and no heart murmur was identified. The examinations of hemogram, urine, and blood biochemical analyses were within normal ranges.

Echocardiography showed irregular-shaped occupying lesions measured 5.4 cm × 3.7 cm in posterior of atrial septum (mainly in left atrium), left heart dilatation with left ventricular systolic dysfunction, medium tricuspid regurgitation, and mild mitral regurgitation. The clinical impression was most compatible with a left atrial myxoma. The patient underwent thoracotomy to excise the mass. At surgery, a globular solid mass was seen arising from the posterior aspect of left atrium. The lesion pushed pulmonary vein to the anterior and compressed inferior vena cava and infiltrated into coronary vein sinus. The enlargement of the right atrium and the right ventricle were observed and the pericardium was normal. Eight days after surgery, ultrasound showed that the patient had massive pericardial effusion. No adjuvant chemotherapy was performed for the patient. Postoperative evaluation did not reveal stigmata or family history of tuberous sclerosis complex (TSC). The patient was discharged after recovered from pericardial effusion. The patient was regularly followed up for 32 months and was doing well.

### 3.2. Case  2

The patient was a 33-year-old African American female with no family history of tuberous complex. She first presented with left atrial mass and that was resected. 8 months later, she underwent a more extensive excision of recurrence and radiation therapy. 2 years later, the patient was found to have a large retroperitoneal mass involving the liver with extension into the inferior vena cava to right atrium. The mass also encased the right kidney. The patient underwent an en bloc resection. 

## 4. Pathology

### 4.1. Gross

#### 4.1.1. Case  1

**Figure 1:**
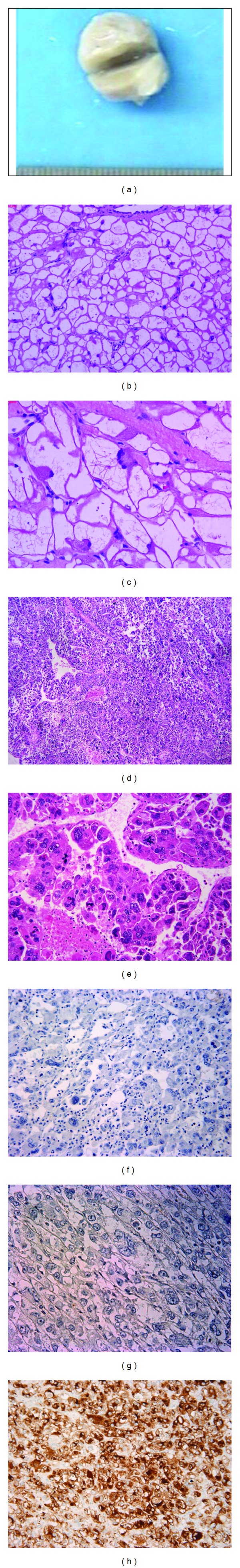
Epithelioid PEComas in hearts. Figures 1(a)–1(c): Case 1. (a) gross picture for case 1. (b)-(c) H&E for case 1 ((b) ×100; (c) ×200); Figures 1(d)–1(h). Case 2: metastasis. (d) tumor with necrosis, H&E, ×50; (e) multinuclear giant cells with nuclear atypia and atypical mitosis, H&E, ×200; (f) case 2, negative cytokeratin (AE1/3) stain, ×200; (g) case 2, negative S100 stain, ×200; (h) case 2, diffusely positive HMB-45 stain, ×200.

The excised globular mass measured 6.0 cm × 4.4 cm × 3.5 cm and had a firm and friable consistency (Figure 1(a)), and was covered by smooth, glistening endocardium, except for the resection surface at the site of attachment. The cut surface was pale yellow with a vague fasciculation.

#### 4.1.2. Case  2

The patient underwent en bloc resection for the retroperitoneal metastasis. The specimen measured 25.0 × 22.5 × 19.0 cm, was tan-white and included portion of right liver, right kidney, and inferior vena cava. A well-circumscribed subcapsular mass in the liver, measuring 5.5 × 4.5 × 4.5 cm was identified in the resected portion of liver. Pathological examination showed that the tumor encasing and invading into inferior vena cava, invasive into the liver and impinged upon but did not invade the kidney. The patient was alive with disease at 33-month status postinitial cardiac surgery.

### 4.2. Microscopic Findings

The histologic features for the two cases were summarized in Table 2. For case 1, the tumor was composed of sheets of large epithelioid cells with striking clear cytoplasm with rod-like eosionophilic cytoplasmic inclusions in some. (Figures 1(b) and 1(c)). The nuclei of the cells are mostly bland and small located at the periphery or center. Scatter cells show moderate nuclear atypia and a few multinuclear giant cells are present. Scant intranuclear pseudoinclusions were occasionally seen in case 1. Focal necrosis was present and mitotic count was 25/50 HPF. The tumor also showed infiltrative growth into native myocardium. For case 2, the tumor was composed of epithelioid cells with eosinophilic cytoplasm and large hyperchromatic nuclei. Coagulation necrosis is seen (Figure 1(d)). Mitotic activity is high and counted for 46 per 50 HPF with atypical mitotic figure. Multinuclear giant cells were numerous (Figure 1(e)). 

### 4.3. Immuhistochemistry

Both of the tumors showed extensive positivity for HMB45 (Figure 1(h)), focal to diffuse positivity for SMA, negative for cytokeratin (Figure 1(f)) and S100 (Figure 1(g), Table 3).

## 5. Discussion

PEComa is a tumor composed of epithelioid cells with clear and acidophilic cytoplasm and having a perivascular distribution. PEComas can occur in a wide variety of sites such as kidney, lung, liver, pancreas, common bile duct, jejunum, rectum, falciform ligament of the liver, urinary bladder, prostate, breast, bone, soft tissue, and female genital tract organs. PEComas occurring in kidney are also termed angiomyolipoma (AML). One rare variant of AML is epithelioid AML and is defined as tumor composed of predominantly epithelioid cells by World Health Organization [18]. Here we borrow that definition and refer to the epithelioid tumors occurring other than kidney as “epithelioid variant of PEComa.” Epithelioid variant of PEComa is extremely rare and is considered potentially malignant with metastasis in one-third of cases in the literature [19]. Nese et al. [19] analyzed 41 cases of pure EAML and found five features associated with adverse prognosis, including history of associated tuberous sclerosis complex or concurrent angiomyolipoma, the presence of necrosis, extrarenal extension and renal vein involvement, large tumor size (>7 cm), and carcinoma-like growth pattern. It was proposed to stratify the tumors into 3 risk groups to predict disease progression, including low risk with tumors having <2 (15% have disease progression); intermediate risk (64% having disease progress) having 2-3 adverse prognostic parameters and high risk (all with disease progression) having four or more adverse prognostic factors. Folpe et al. [20] proposed criteria for the classification of non-AML PEComas. PEComas of <5 cm in size with low cellularity, low nuclear grade, mitotic rate less than 1/50 high power field, and lack of infiltration, necrosis, and vascular invasion should be considered benign. Tumors that show 2 or more of the following features: size greater than 5 cm, infiltrative growth pattern, high mitotic rate, pleomorphism, necrosis, or vascular invasion are considered to be malignant, whereas those with only a single atypical feature are regarded as having uncertain malignant potential. But firm criteria for malignancy of PEComas remain uncertain as its rarity. 

The cardiac PEComas are very rare. To date, only 5 cases of cardiac PEComas were reported [10, 14–17]. The clinicopathologic features of those cases were summarized in Table 4. In this study, we present two cases of pure epithelioid PEComas. In the total of 7 cases (5 reported in the English literature, 2 case from this report) four are from left atrium, 2 from right atrium, one from pericardium. The patient age ranges from 2–48 years old with an average of 26 years of age. There are 5 females and 2 males. Five patients' clinical information regarding TSC histories are available, all of them are negative. One of our cases showed severe nuclear atypia, brisk mitosis, and necrosis with extensive metastasis to retroperitoneal and liver. Although the number is small, three cases including 2 cases from literature and one current case with aggressive behaviors show epithelioid morphology suggesting the cardiac epithelioid PEComas can behave aggressively, likely even more so than their counterpart in other parts of the body due to its cardiac location. 

The main differential diagnosis for cardiac PEComas is cardiac myxoma clinically and rhabdomyoma histologically, especially in children. Rhabdomyomas are the most common pediatric heart tumors and considered as hamartomas with no malignant potential. Cardiac rhabdomyoma is highly associated with tuberous sclerosis complex (TSC). Morphologically, cardiac rhabdomyomas are well demarcated nodules of enlarged cardiac myocytes with cleared cytoplasm. In some cells, strands of eosinophilic cytoplasm stretch from a central nucleus to the cell membrane, giving rise to cells that resemble a spider (“spider cell”). The majority of cells show vacuolization with sparse myofilaments. There is a strong reaction with periodic acid-Schiff reagent, reflecting the glycogen content of rhabdomyoma cells. Immunohistochemical studies show the striated muscle characteristics of rhabdomyoma cells, which express myoglobin, desmin, and actin. Moreover, it has been reported that aberrant HMB45 expression was detected in cardiac rhabdomyomas [21]. Nevertheless, PEComas should have smooth muscle differentiation such as SMA reactivity rather than striated muscle differentiation such as myoglobin reactivity. The striking clear cell features in our second case is very similar to that of the sugar tumor of the lung, a member of the PEComa family. Cardiac epithelioid PEComa with clear cell features needs to be differentiated from metastatic tumors with clear cell features from various primary sites such as kidney, skin, and adrenal.

Because of their rarity, optimal treatment for PEComas is not determined. Surgical resection represents the only curative approach for primary PEComa as well as for local recurrences and metastasis, as chemotherapy and radiotherapy have not demonstrated significant benefits [22, 23].

In summary, we have documented herein two cases of epithelioid PEComas in the heart, one of which developed extensive metastasis. Cardiac PEComa tends to occur in young adult and pediatric patients with slightly predilection to the left atrium and female gender. Necrosis is common features in cardiac PEComa and presence of nuclear atypia and high mitotic activity could be associated with aggressive behaviors. Additional cases and longer followup are needed to gain more insight into tumor biologic behavior in those cases lacking marked nuclear atypia and high mitotic activity.

## Figures and Tables

**Table 1:** Antibodies.

Antibody	Species	Working dilution	Supplier
AE1-3	Mouse/monoclonal	1 : 400	Novocastra
HMB45	Mouse/monoclonal	1 : 40	Neomarkers
SMA	Mouse/monoclonal	1 : 600	Dako
S100	Cow/polyclonal	1 : 3750	Dako

**Table 2:** Clinical features of current two cases.

Case	Age (years)	Gender	site	Size (cm)	Metastasis (organ, tumor size (cm))
1	2	F	Left atrium	6	no
2	33	F	Left atrium	Unknown	25, liver and retroperitoneum

**Table 3:** Histological characteristics of current two cases.

Case	Necrosis	Multinuclear giant cells	Nuclear atypia	Mitosis (50 HPF)	Atypical mitosis	Vascular invasion
1	Yes	Yes	Mild	25	No	No
2	Yes	Yes	Severe	46	Yes	Yes

**Table 4:** Summary of clinicopathologic features of 7 reported cardiac PEComas.

Case	Age	Sex	TSC	Site	Size (cm)	Morphology	Mitosis/50 HPF	Atypical mitosis	Necrosis	Followup (month)
1 [14]	48	F	No	Rt atrium	6	Mixed vessels, smooth muscle, and fat	No	No	No	A&W, 20
2 [15]	38	F	No	Rt atrium	34	Mixed vessels, smooth muscle, and fat	Rare	—	—	Died, 9 days post-op
3 [10]	29	M	—	Lt atrium	12	Epithelioid cells	45	Yes	Yes	Died, post-op (not tumor related)
4 [16]	20	M	—	Pericardium	15	Epithelioid cells	>1	NA	Yes	A&W, 6
5 [17]	10	F	No	Lt atrioventricular groove	6	Spindle and epithelioid cells with bland nuclei	Rare	—	No	A&W, 18
6*	2	F	No	Lt atrium	6	Epithelioid cells	25/HPF	No	Yes	A&W, 32
7*	33	F	No	Lt atrium	NA	Epitheliod cells	46/HPF	Yes	Yes	AWD, 36

A&W: alive and well; post-op: postoperation; AWD: alive with disease; *: our cases.
